# (±)-2-Oxocyclo­penta­neacetic acid: catemeric hydrogen bonding in a γ-keto acid

**DOI:** 10.1107/S1600536809010708

**Published:** 2009-03-31

**Authors:** Georgia Efthimiopoulos, Markos M. Papadakis, Hugh W. Thompson, Roger A. Lalancette

**Affiliations:** a Carl A. Olson Memorial Laboratories, Department of Chemistry, Rutgers University, Newark, NJ 07102, USA

## Abstract

The title racemate, C_7_H_10_O_3_, aggregates in the solid as acid-to-ketone hydrogen-bonding catemers [O⋯O = 2.7050 (13) Å and O—H⋯O = 166.1 (17)°] having glide-related components. Four such heterochiral chains, paired centrosymmetrically about (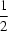, 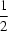, 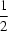) in the cell, proceed through the cell in the 010 direction, with alignment with respect to the *c* axis of ++−−.

## Related literature

For background to catemers and hydrogen bonds, see: Barcon *et al.* (1998 ▶, 2002 ▶); Coté *et al.* (1996 ▶); DeVita Dufort *et al.* (2007 ▶); Efthimiopoulos *et al.* (2009 ▶); Harata *et al.* (1977 ▶); Lalancette & Thompson (2003 ▶); Lalancette *et al.* (2006 ▶); Malak *et al.* (2006 ▶); Newman *et al.* (2002 ▶); Steiner (1997 ▶); Stork *et al.* (1963 ▶).
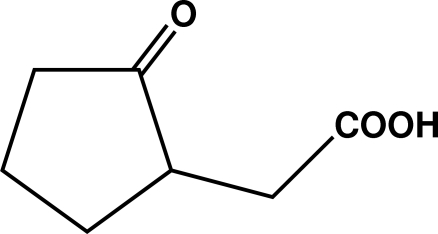


## Experimental

### 

#### Crystal data

C_7_H_10_O_3_
*M* *_r_* = 142.15
Orthorhombic, 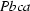

*a* = 5.3232 (1) Å
*b* = 12.2981 (3) Å
*c* = 20.8148 (5) Å
*V* = 1362.65 (5) Å^3^
*Z* = 8
Cu *K*α radiation
μ = 0.91 mm^−1^
*T* = 100 K
0.37 × 0.15 × 0.10 mm

#### Data collection

Bruker SMART CCD APEXII area-detector diffractometer
Absorption correction: multi-scan (*SADABS*; Sheldrick, 2008*a* ▶) *T*_min_ = 0.730, *T*_max_ = 0.915
9810 measured reflections
1197 independent reflections
1147 reflections with *I* > 2σ(*I*)
*R*_int_ = 0.016

#### Refinement

*R*[*F* ^2^ > 2σ(*F* ^2^)] = 0.033
*wR*(*F* ^2^) = 0.086
*S* = 1.04
1197 reflections
95 parameters
H atoms treated by a mixture of independent and constrained refinement
Δρ_max_ = 0.26 e Å^−3^
Δρ_min_ = −0.15 e Å^−3^

### 

Data collection: *APEX2* (Bruker, 2006 ▶); cell refinement: *SAINT* (Bruker, 2006 ▶); data reduction: *SAINT*; program(s) used to solve structure: *SHELXTL* (Sheldrick, 2008*b* ▶); program(s) used to refine structure: *SHELXTL*; molecular graphics: *SHELXTL*; software used to prepare material for publication: *SHELXTL*.

## Supplementary Material

Crystal structure: contains datablocks I, global. DOI: 10.1107/S1600536809010708/fl2241sup1.cif

Structure factors: contains datablocks I. DOI: 10.1107/S1600536809010708/fl2241Isup2.hkl

Additional supplementary materials:  crystallographic information; 3D view; checkCIF report

## Figures and Tables

**Table 1:** Hydrogen-bond geometry (Å, °)

*D*—H⋯*A*	*D*—H	H⋯*A*	*D*⋯*A*	*D*—H⋯*A*
O3—H3⋯O1^i^	0.832 (19)	1.890 (19)	2.7050 (13)	166.1 (17)

Symmetry code: (i) 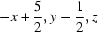.

**Fig. 1.**
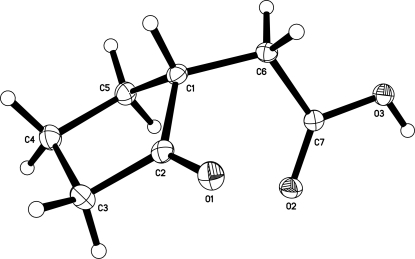
A view of the asymmetric unit of (I) with its numbering scheme. Displacement ellipsoids are drawn at the 40% probability level for non-H atoms.

**Fig. 2.**
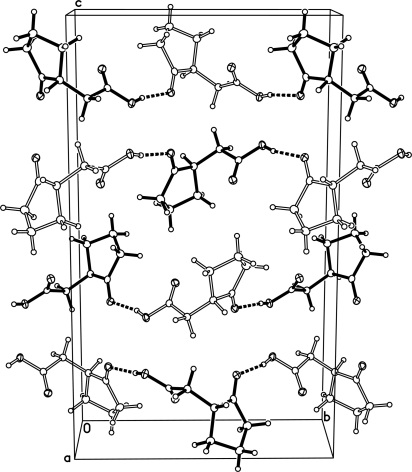
A packing diagram, illustrating the four heterochiral catemers created by acid-to-ketone H bonds proceeding along chains of molecules glide-related in the 010 direction. The handedness of the molecules is differentiated by the shading of the bonds. Starting at the origin, the order of the directional alignment of the four chains with respect to the c axis is + + - -. Displacement ellipsoids are drawn at the 40% probability level for non-H atoms.

## supplementary crystallographic information

### Comment

Our study of the crystal structures of ketocarboxylic acids explores their five known H-bonding modes. Two of these do not involve the ketone, corresponding to the common pairing and much rarer chain modes in simple acids. Acid-to-ketone chains (catemers) constitute a sizable overall minority of cases, while acid-to-ketone dimers and intramolecular H-bonds are rarely observed. We have presented examples of many of these and have discussed factors that contribute to the choice of mode (Coté *et al.*, 1996; Newman *et al.*, 2002; Lalancette *et al.*, 2006; DeVita Dufort *et al.*, 2007).

An issue of interest is the minimum requirements for catemer formation. However, the very smallest molecules offer several experimental problems (volatility, low crystallinity, little structural variability), so that few C_3_—C_6_ keto-acids have been previously reported (Harata *et al.*, 1977; Malak *et al.*, 2006; Efthimiopoulos *et al.*, 2009). We now report the crystal structure of the title C_7_γ-keto acid (I), among the smallest found to aggregate in the solid as a catemer. The category of γ-keto acids is especially rich in H-bonding types, embracing internal H bonds and catemers of the screw, translation and glide types, as well as dimers and hydrated patterns. The intra-chain glide relationship found is considerably rarer than either screw or translational schemes generally, and is shared with three other γ-keto acids of our experience (Barcon *et al.*, 1998, 2002; DeVita Dufort *et al.*, 2007).

Fig. 1 presents a view of the asymmetric unit of (I) with its numbering. The conformation adopted by the ring involves flexing of the two ring-carbons most remote from the ketone, C4 & C5, so as to place them farthest from the average ring-plane, -0.2218 (9) & 0.2424 (9) Å, respectively, on opposite faces of the ring. This permits staggering of all ring-H atoms and projects the side-chain pseudo-equatorially, with a O1—C2—C1—C6 torsion angle of 32.63 (17)°.

In solution, full rotation about both C—C bonds in the side-chain is possible; however, in the solid the staggering requirements about C1—C6 allow few real options. The observed C2—C1—C6—C7 torsion angle of 57.31 (14)° places the carboxyl group maximally away from the ring plane, and the carboxyl is rotated so that its carbonyl is essentially coplanar with the C1—C6 bond [O2—C7—C6—C1 = -7.31 (18)°]. The intramolecular dihedral angle between the carboxyl and ketone planes is 75.03 (5)°.

Averaging of C—O bond lengths and C—C—O angles by disorder, although common in carboxyl dimers, is not seen in acid-to-ketone catemers, whose geometry cannot support any of the averaging mechanisms required. In (I) these C—O bonds have lengths of 1.2082 (15) & 1.3292 (15) Å, with angles of 125.09 (11) & 110.72 (10)°, similar to those in other fully ordered carboxylic acids.

Fig. 2 shows the packing of the cell and the parallel carboxyl-to-ketone H-bonding chains, all passing through the cell in the 010 direction. The chain components are glide-related with O···O distances of 2.7050 (13) Å, and O—H···O angles of 166.1 (17)°. The intermolecular dihedral angle for the acid *versus* ketone planes is 29.04 (8) °. Among H-bonding catemers, the observed prevalence of subtypes, describing the relation of adjacent molecules, is homochiral (screw > translation) > heterochiral (glide). The four heterochiral H-bonding chains in (I) are paired centrosymmetrically about 1/2, 1/2, 1/2, with each enantiomer in the array appearing four times. Starting at the origin, the order of the directional alignment of the four chains with respect to the *c* axis is + + - -.

We characterize the geometry of H bonding to carbonyls by a combination of H···O=C angle and H···O=C—C torsion angle. These describe the approach of the acid H-atom to the O in terms of its deviation from, respectively, C=O axiality (ideal = 120°) and planarity with the carbonyl (ideal = 0°). In (I) the values for these two angles are 125.2 (5) & -10.2 (7)°. No intermolecular C—H···O contacts were found within the 2.6 Å range we routinely survey for such close non-bonded polar interactions (Steiner, 1997).

Among the factors disfavoring standard dimeric carboxyl H bonding, we have identified low availability of alternative conformations. The conformational flexibility associated with cyclopentane rings is a solution characteristic; in the crystal, the requirements disfavoring hydrogen eclipsing and favoring pseudo-equatorial substituents leaves a system like (I) with few actual conformational options. As a result (I) joins a number of nominally flexible cyclic molecules we have found that behave much more like rigid systems and adopt catemeric H-bonding modes (Barcon *et al.*, 2002; Malak *et al.*, 2006; Lalancette & Thompson, 2003).

Because of the similar shifts produced by ketone ring-strain and by H bonding, the solid-state *versus* liquid IR spectra of carboxycyclopentanones are typically ambiguous regarding H bonding in the crystal. The solid-state (KBr) spectrum of (I) has C=O stretching absorptions at 1735 (acid) and 1721 cm^-1^ (ketone), consistent with known shifts produced when H-bonding is removed from carboxyl C=O and added to a ketone. In CHCl_3_ solution these peaks appear, presumably reversed, at 1736 and 1714 cm^-1^.

### Experimental

The ethyl ester of 2-oxocyclopentaneacetic acid, prepared *via* the enamine (Stork *et al.*, 1963), was hydrolyzed by refluxing with conc. HCl. Distilled keto acid was recrystallized from ether-hexane to give material suitable for X-ray, mp 327 K.

### Refinement

All H atoms for (I) were found in electron density difference maps. The carboxyl H was refined positionally with *U*_iso_(H) = 1.5*U*_eq_(O). The methylene and methine Hs were placed in geometrically idealized positions and constrained to ride on their parent C atoms with C—H distances of 0.99 and 1.00 Å, respectively, and *U*_iso_(H) = 1.2*U*_eq_(C).

### Crystal data

**Table:**

C_7_H_10_O_3_	*D*_x_ = 1.386 Mg m^−^^3^
*M**_r_* = 142.15	Melting point: 327 K
Orthorhombic, *P**b**c**a*	Cu *K*α radiation, λ = 1.54178 Å
Hall symbol: -P 2ac 2ab	Cell parameters from 7778 reflections
*a* = 5.3232 (1) Å	θ = 4.3–67.1°
*b* = 12.2981 (3) Å	µ = 0.91 mm^−^^1^
*c* = 20.8148 (5) Å	*T* = 100 K
*V* = 1362.65 (5) Å^3^	Needle, colourless
*Z* = 8	0.37 × 0.15 × 0.10 mm
*F*(000) = 608	

### Data collection

**Table:**

Bruker SMART CCD APEXII area-detector diffractometer	1197 independent reflections
Radiation source: fine-focus sealed tube	1147 reflections with *I* > 2σ(*I*)
graphite	*R*_int_ = 0.016
φ and ω scans	θ_max_ = 67.0°, θ_min_ = 4.3°
Absorption correction: multi-scan (*SADABS*; Sheldrick, 2008a)	*h* = −6→6
*T*_min_ = 0.730, *T*_max_ = 0.915	*k* = −14→14
9810 measured reflections	*l* = −24→24

### Refinement

**Table:**

Refinement on *F*^2^	Secondary atom site location: difference Fourier map
Least-squares matrix: full	Hydrogen site location: inferred from neighbouring sites
*R*[*F*^2^ > 2σ(*F*^2^)] = 0.033	H atoms treated by a mixture of independent and constrained refinement
*wR*(*F*^2^) = 0.086	*w* = 1/[σ^2^(*F*_o_^2^) + (0.0444*P*)^2^ + 0.8053*P*] where *P* = (*F*_o_^2^ + 2*F*_c_^2^)/3
*S* = 1.04	(Δ/σ)_max_ < 0.001
1197 reflections	Δρ_max_ = 0.26 e Å^−^^3^
95 parameters	Δρ_min_ = −0.15 e Å^−^^3^
0 restraints	Extinction correction: *SHELXTL* (Sheldrick, 2008b), Fc^*^=kFc[1+0.001xFc^2^λ^3^/sin(2θ)]^-1/4^
Primary atom site location: structure-invariant direct methods	Extinction coefficient: 0.0014 (4)

### Special details

**Table:**

Experimental. Crystal mounted on a Cryoloop using Paratone-N
Geometry. All e.s.d.'s (except the e.s.d. in the dihedral angle between two l.s. planes) are estimated using the full covariance matrix. The cell e.s.d.'s are taken into account individually in the estimation of e.s.d.'s in distances, angles and torsion angles; correlations between e.s.d.'s in cell parameters are only used when they are defined by crystal symmetry. An approximate (isotropic) treatment of cell e.s.d.'s is used for estimating e.s.d.'s involving l.s. planes.
Refinement. Refinement of *F*^2^ against ALL reflections. The weighted *R*-factor *wR* and goodness of fit *S* are based on *F*^2^, conventional *R*-factors *R* are based on *F*, with *F* set to zero for negative *F*^2^. The threshold expression of *F*^2^ > σ(*F*^2^) is used only for calculating *R*-factors(gt) *etc*. and is not relevant to the choice of reflections for refinement. *R*-factors based on *F*^2^ are statistically about twice as large as those based on *F*, and *R*- factors based on ALL data will be even larger.

### Fractional atomic coordinates and isotropic or equivalent isotropic displacement parameters (Å^2^)

**Table:**

	*x*	*y*	*z*	*U*_iso_*/*U*_eq_	
O1	1.12228 (17)	0.12579 (7)	0.31120 (4)	0.0196 (3)	
C1	0.7561 (2)	0.03606 (10)	0.35627 (6)	0.0156 (3)	
H1	0.6138	0.0786	0.3375	0.019*	
C2	0.9814 (2)	0.11135 (10)	0.35642 (6)	0.0156 (3)	
O2	1.16880 (17)	−0.11081 (7)	0.37021 (4)	0.0205 (3)	
O3	1.00596 (18)	−0.22599 (7)	0.29778 (4)	0.0193 (3)	
H3	1.131 (4)	−0.2640 (14)	0.3061 (8)	0.029*	
C3	0.9997 (2)	0.16595 (11)	0.42143 (6)	0.0209 (3)	
H3A	1.0087	0.2460	0.4167	0.025*	
H3B	1.1505	0.1407	0.4449	0.025*	
C4	0.7603 (3)	0.13271 (11)	0.45681 (6)	0.0213 (3)	
H4A	0.6238	0.1858	0.4492	0.026*	
H4B	0.7905	0.1268	0.5036	0.026*	
C5	0.6948 (2)	0.02124 (11)	0.42777 (6)	0.0192 (3)	
H5A	0.7984	−0.0371	0.4470	0.023*	
H5B	0.5150	0.0037	0.4341	0.023*	
C6	0.7852 (2)	−0.06445 (10)	0.31485 (6)	0.0154 (3)	
H6A	0.6307	−0.1088	0.3186	0.018*	
H6B	0.8010	−0.0415	0.2694	0.018*	
C7	1.0077 (2)	−0.13453 (10)	0.33180 (6)	0.0142 (3)	

### Atomic displacement parameters (Å^2^)

**Table:**

	*U*^11^	*U*^22^	*U*^33^	*U*^12^	*U*^13^	*U*^23^
O1	0.0220 (5)	0.0169 (5)	0.0198 (5)	−0.0025 (4)	0.0027 (4)	0.0010 (3)
C1	0.0140 (6)	0.0151 (6)	0.0176 (6)	0.0018 (5)	−0.0010 (5)	0.0005 (5)
C2	0.0164 (6)	0.0123 (6)	0.0180 (6)	0.0026 (5)	−0.0013 (5)	0.0027 (5)
O2	0.0191 (5)	0.0196 (5)	0.0228 (5)	0.0020 (4)	−0.0054 (4)	−0.0024 (4)
O3	0.0194 (5)	0.0143 (5)	0.0241 (5)	0.0027 (4)	−0.0031 (4)	−0.0034 (3)
C3	0.0230 (7)	0.0206 (7)	0.0192 (7)	−0.0038 (5)	−0.0008 (5)	−0.0026 (5)
C4	0.0227 (7)	0.0220 (7)	0.0191 (7)	0.0001 (5)	0.0015 (5)	−0.0034 (5)
C5	0.0182 (6)	0.0200 (6)	0.0192 (6)	−0.0015 (5)	0.0036 (5)	−0.0013 (5)
C6	0.0148 (6)	0.0150 (6)	0.0163 (6)	−0.0014 (5)	−0.0011 (5)	0.0002 (5)
C7	0.0150 (6)	0.0134 (6)	0.0141 (6)	−0.0026 (5)	0.0028 (5)	0.0020 (4)

### Geometric parameters (Å, °)

**Table:**

O1—C2	1.2165 (15)	C3—H3A	0.9900
C1—C2	1.5150 (17)	C3—H3B	0.9900
C1—C6	1.5150 (16)	C4—C5	1.5383 (18)
C1—C5	1.5345 (17)	C4—H4A	0.9900
C1—H1	1.0000	C4—H4B	0.9900
C2—C3	1.5138 (17)	C5—H5A	0.9900
O2—C7	1.2082 (15)	C5—H5B	0.9900
O3—C7	1.3292 (15)	C6—C7	1.5066 (17)
O3—H3	0.832 (19)	C6—H6A	0.9900
C3—C4	1.5276 (18)	C6—H6B	0.9900
			
C2—C1—C6	114.76 (10)	C3—C4—H4B	111.0
C2—C1—C5	103.82 (10)	C5—C4—H4B	111.0
C6—C1—C5	118.47 (10)	H4A—C4—H4B	109.0
C2—C1—H1	106.3	C1—C5—C4	103.12 (10)
C6—C1—H1	106.3	C1—C5—H5A	111.1
C5—C1—H1	106.3	C4—C5—H5A	111.1
O1—C2—C3	125.94 (11)	C1—C5—H5B	111.1
O1—C2—C1	125.14 (11)	C4—C5—H5B	111.1
C3—C2—C1	108.91 (10)	H5A—C5—H5B	109.1
C7—O3—H3	111.1 (12)	C7—C6—C1	114.45 (10)
C2—C3—C4	104.97 (10)	C7—C6—H6A	108.6
C2—C3—H3A	110.8	C1—C6—H6A	108.6
C4—C3—H3A	110.8	C7—C6—H6B	108.6
C2—C3—H3B	110.8	C1—C6—H6B	108.6
C4—C3—H3B	110.8	H6A—C6—H6B	107.6
H3A—C3—H3B	108.8	O2—C7—O3	124.18 (11)
C3—C4—C5	103.78 (10)	O2—C7—C6	125.09 (11)
C3—C4—H4A	111.0	O3—C7—C6	110.72 (10)
C5—C4—H4A	111.0		
			
C6—C1—C2—O1	32.63 (17)	C2—C1—C5—C4	34.53 (12)
C5—C1—C2—O1	163.49 (12)	C6—C1—C5—C4	163.15 (11)
C6—C1—C2—C3	−147.97 (11)	C3—C4—C5—C1	−39.41 (12)
C5—C1—C2—C3	−17.11 (13)	C2—C1—C6—C7	57.31 (14)
O1—C2—C3—C4	172.13 (12)	C5—C1—C6—C7	−66.02 (14)
C1—C2—C3—C4	−7.26 (13)	C1—C6—C7—O2	−7.31 (18)
C2—C3—C4—C5	28.73 (13)	C1—C6—C7—O3	173.81 (10)

### Hydrogen-bond geometry (Å, °)

**Table:**

*D*—H···*A*	*D*—H	H···*A*	*D*···*A*	*D*—H···*A*
O3—H3···O1^i^	0.832 (19)	1.890 (19)	2.7050 (13)	166.1 (17)

Symmetry codes: (i) −*x*+5/2, *y*−1/2, *z*.
